# Effects of Medicine on the Human System

**Published:** 1854-11

**Authors:** E. C. Atkinson

**Affiliations:** Dover, Iowa


### EFFECTS OF MEDICINE ON THE HEALTHY HUMAN SYSTEM.

BY E. C. ATKINSON, M. D., DOVER, IOWA.

The science of therapeutics has now attained a degree of perfection at least equal to that of any other. The prescriptions of the physician, who merits the name, are no longer influenced by magical incantations, and visionary notions of relations and affinities which never existed. His opportunities enable him to acquire a comprehension of the relations of medicines to each other, and to diseased action, so that he can rest his judgment on the legitimate basis of sound philosophy. After determining the pathology of the case before him, the experienced practitioner may generally prescribe with singular confidence as to the effect which will follow, often eradicating disease in its incipiency, controlling it in its culmination and decline, and alleviating what it cannot cure.

With the physiological effects of medicine, however, we are not so well acquainted. The science of toxicology is yet in its infancy. This arises not only from the fact that it is a subject of comparatively modern inquiry, but from the limited field of observation to which the student of this department of science is necessarily restricted.

While he may have daily opportunities of witnessing the effect of curative agents in disease, he is indebted chiefly to the occasional circumstance of accident, or criminal design, for testing their effects in health. Nor is this the only difficulty he has to encounter ; under the same modifying influences, the action of medicine is less uniform in the healthy than in the diseased state.

When disease invades the system, it levels to a great extent its peculiar sympathies and idiosyncrasies, and establishes a tolerance of the agents necessary for its cure. In health, on the other hand, with all its sympathies in full play, each system is acted upon differently. Thus we might give a certain quantity of Tinct. opii to a number of individuals of the same nativity, and apparently of the same condition; but while we should find the lives of some destroyed by its effect, others would hardly be in the least affected; a fact not referable to their muscular strength, but to a different susceptibility of their nervous systems to its action.

Again, medicines sometimes exhibit a scpecific effect on the healthy system, to a knowledge of which their effect on the same system in disease furnishes us no precedent. An interesting illustration of this principle recently came under my notice.

An old lady of much experience as a nurse, asked me for a mild laxative, cautioning me at the same time not to give her rhubarb, for said she, "it always gives me violent strangury." A few weeks subsequently she called on me again for a similar prescription. I gave her rhubarb concealed in pills; a severe strangury was the result, which, however, was soon relieved. A few months after she suffered from a severe attack of erysipelas, during the course of which she was troubled by a persistent watery diarrhœa, for which I gave her rhubarb at several different times, with no other than the best effects. Since her recovery, I have been informed of another case on whom rhubarb produced the same bad result. With these facts before us, it is obvious that some time must elapse before Toxicology can be considered a matured science resting on a firm foundation. The task, however, by the energy of the medical profession, will doubtless in due time be accomplished.

Without further preliminary, I submit the following record of cases for publication, without attempting a philosophical analysis of the facts which they contain.

*Effects of Opium.*—Case I.—Mrs. C., aged 69, never had much sickness, intellectual faculties but little impaired, was advised by a friend to take Tinct. opii for a slight diarrhœa, accordingly took 30 gtt. on going to bed. About fifteen minutes after retiring, her moanings awakened her daughter, who found her in great distress, though sufficiently rational to inform her what she had taken. Her daughter at once sent for medical aid, but before I arrived she was dead. I ascertained that about fifteen minutes after the toxical effects were first exhibited, she sank into a perfect cóma, and in about two hours died.

Case II.—Mr. B., aged 18 years, on account of some difficulty with his lady-love, attempted suicide by taking of Tinct. opii 20 gtt. This small quantity, however, produced an extreme impression. After taking it, he retired to his room. One hour after, when found, the following symptoms were presented: pulse moderately full, beating but 40 per minute, stertorous respiration, countenance livid and suffused, skin moist and cool, extreme stupor, total insensibility to external impressions, and powers of life sinking. After trying a variety of means for his relief, with no apparent good effect, we resorted to showering his head with cold water, under the influence of which he gradually recovered, though after he was revived, there was for some hours a great tendency to relapse, requiring the most vigilant care, and repeated applications of the water.

Case III.—G., a child of one week old, strong and healthy—during the night its mother gave it 8 gtt. of laudanum to keep it quiet. It sank into a stupor, in which state it remained for a space of nearly ten hours, after which, by the use of energetic means, it was aroused, and slowly recovered; during convalescence it had several severe convulsions, from which it was completely restored.

Case IV.—C., a child seven days old, large and healthy, its mother gave it 7 gtt. Tinct. opii in the night to keep it quiet; in about an hour it sank into a stupor. At the end of about two hours I was called in. It then presented all the symptoms of extreme narcotism. Notwithstanding our utmost efforts, it died in about eight hours; it revived several times, and we fondly hoped the worst was over, but the nervous system had received an impression incompatible with life. The closing symptoms were severe convulsions. I shall ever regret that I did not make use of artificial respiration, the only remedy which I think might have been of benefit. The practice of giving laudanum to very young infants, either in health or disease, cannot be too severely reprobated. I have met with several cases of convulsions in infants, under one year old, caused, I have reason to suppose, by the secondary effects of some form of opium.

Case V.—Mr. A., a young man, aged 20 years, of nervous temperament, good habits and good constitution, while suffering from toothache, was advised by a careless physician to take laudanum.

He accordingly purchased an ounce, and being ignorant of its nature took three-eighths of it. After taking it, he started for his home, five miles distant, and with great effort reached it, retired immediately to his room, and slept ten hours. At the end of this time I saw him, all the symptoms of narcotism were passing off, and in two days he was well.

I once heard a physician of reputation and experience testify that three drachms of laudanum retained on the stomach of an adult would "in all cases as a general rule produce death." Had he been well acquainted with the different works on Toxicology, he would have known that this rule has too many exceptions to be depended on. Equally as unintelligent testimony is frequently given by physicians in medicolegal cases to the great disgrace of our profession.

*Effects of Morphia.*—Case I.—C., a boy two years old, took one-sixth of a grain of sulph. morphia, through mistake for calomel. In about one hour he was seized with opisthotonos, which continued about five hours, when the life powers suddenly yielded. Some little stupor was present prior to the attack coming on.

I never have known this effect to follow a narcotic in any other instance. In this case it must have been produced by the morphia, as there was no other cause for it.

*Effect of Camphor.*—Case I.—L., a man aged 45, an inveterate drunkard, during a debauch, drank about one and a half ounces of saturated tinct. camphor, was seized with convulsions one hour afterwards, and died in forty minutes.

Case II.—Mr. A., a young man aged 21 years, while walking the street with a friend, *accidentally* swallowed one drachm of gum camphor. Nothing more was thought of it, until one hour after, when he suddenly dropped to the ground while talking, and remained in a complete stupor for about half an hour. He then revived and resumed conversation as if nothing had happened—no medication was resorted to.

I have on record three other similar cases; complete anæsthesia was the result in each, and no bad consequences ensued.

Case III.—Mrs. K., aged 34. Five powders of camphor of three grains each, pulverised by addition of a few drops of Spts. Nit. dulc., were left her for after pains; after taking the third, convulsions supervened, though mild, and soon passed off; on the next day the other two were given with like effect. Some authors state that camphor produces convulsions only when taken in solution, a rule which evidently has exceptions.

*Sulphate of Zinc.*—Case 1.—D., a young man aged 28, took one table-spoonful of sulphate of zinc, supposing it to be Epsom Salts; violent vomiting, and subsequently violent purging ensued. These effects soon terminated spontaneously, and on the next day he resumed his business. I examined the sulphate of zinc, and found it good article, slightly damaged by contact with the atmosphere.

